# Systematic Activity Maturation of a Single-Domain Antibody with Non-canonical Amino Acids through Chemical Mutagenesis

**DOI:** 10.1016/j.chembiol.2020.11.002

**Published:** 2021-01-21

**Authors:** Philip R. Lindstedt, Francesco A. Aprile, Pietro Sormanni, Robertinah Rakoto, Christopher M. Dobson, Gonçalo J.L. Bernardes, Michele Vendruscolo

**Affiliations:** 1 Centre for Misfolding Diseases, Department of Chemistry, University of Cambridge, CB2 1EW Cambridge, UK; 2 Department of Chemistry, Molecular Sciences Research Hub, Imperial College London, London W12 0BZ, UK; 3 Instituto de Medicina Molecular João Lobo Antunes, Faculdade de Medicina, Universidade de Lisboa, 1649-028 Lisboa, Protugal

**Keywords:** antibody maturation, chemical mutagenesis, non-natural amino acids, protein aggregation

## Abstract

Great advances have been made over the last four decades in therapeutic and diagnostic applications of antibodies. The activity maturation of antibody candidates, however, remains a significant challenge. To address this problem, we present a method that enables the systematic enhancement of the activity of a single-domain antibody through the post-translational installation of non-canonical side chains by chemical mutagenesis. We illustrate this approach by performing a structure-activity relationship study beyond the 20 naturally occurring amino acids on a single-domain antibody designed *in silico* to inhibit the aggregation of the amyloid-β peptide, a process closely linked to Alzheimer's disease. We found that this approach can improve, by five orders of magnitude, the anti-aggregation activity of the starting single-domain antibody, without affecting its stability. These results show that the expansion of the chemical space available to antibodies through chemical mutagenesis can be exploited for the systematic enhancement of the activity of these molecules.

## Highlights

• Chemical mutagenesis was pursued along the CDR3 loop of a single-domain antibody
• Sites deemed accessible had diverse side chains screened for activity enhancement
• Final mutant had greatly enhanced activity and maintained other desired properties

## Graphical Abstract

**Figure:**
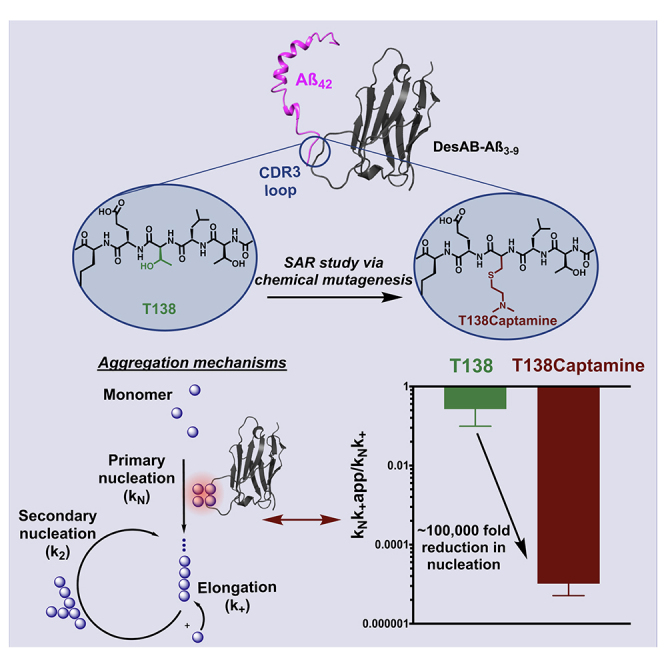

## Teaser

Lindstedt et al. investigated the application of chemical mutagenesis to perform a SAR study on a single-domain antibody. The final chemical mutant had greatly enhanced activity with only one side-chain alteration and maintained other biophysical properties, highlighting the utility of this minimalist approach for protein activity maturation.

## Introduction

Antibodies have become a cornerstone of modern medicine and biotechnology and are increasingly used as therapeutic agents for a wide range of diseases (Carter and Lazar, 2018; Grilo and Mantalaris, 2019; Kaplon et al., 2020). A wide arsenal of technologies is currently utilized for antibody discovery, including immunization and display methods (Boder et al., 2000; Bradbury et al., 2011; Hoogenboom, 2005; Miersch and Sidhu, 2012; Sidhu, 2000; Winter et al., 1994). However, many of these procedures require a significant amount of time and resources for the development of fully functional antibodies. One particular area that has proven difficult for therapeutic antibodies is the maturation of their biological activity while preserving other important properties, such as epitope selectivity, conformational stability, and solubility (Bradbury and Plückthun, 2015; Lerner, 2016; Liu, 2014).

The high-throughput nature of display methods (up to 10^10^ variants in the case of phage display) enables the exploration of the chemical space accessible through the 20 naturally occurring amino acids at the various positions of the antibody-target interface (Sidhu, 2000). While this approach leads, in a variety of cases, to the discovery of effective antibodies, to further expand the scope of antibody applications it would be desirable to be able to perform with these large molecules the traditional structure-activity relationship (SAR) studies typically carried out for small molecules (Cherkasov et al., 2014; Dobson, 2004; Kolb et al., 2001; Tropsha, 2010). SAR studies allow the accurate assessment of the biological effects brought about by small physicochemical changes in the starting structure, optimizing the molecule atom-by-atom thanks to the synthetic power of modern medicinal chemistry (Cherkasov et al., 2014; Guha, 2013).

If the same kind of exquisite chemical control that medicinal chemists have with small molecules could be achieved with the amino acid side chains of antibodies, a similarly rational exploration of the chemical space at key residues along the paratope could enable a direct path to maturing initial candidates. At the same time, it is possible to predict the effects that such small changes would have on other biophysical traits, such as stability, thus offering a more manageable system. While genetic codon expansion technology has been used previously to incorporate unnatural amino acids (UAAs) into antibodies with unique properties, this approach ultimately relies on traditional display methods, and each UAA expression system can only increase the chemical lexicon one residue at a time (Chin et al., 2003; Lang and Chin, 2014). For the chemical space to be efficiently explored, there should ideally be a post-translational system for the rapid and efficient installation of a variety of diverse side chains at a site of interest (Krall et al., 2016; Prescher and Bertozzi, 2005; Wright et al., 2016b).

Here, we report the use of the post-translationally installed synthetically versatile non-canonical amino acid dehydroalanine (Dha) to create a platform for the precise augmentation of the activity of antibodies for inhibiting the aggregation of the 42-residue form of the amyloid-β peptide (Aβ42), a protein fragment closely associated with Alzheimer's disease (AD) (Hardy and Selkoe, 2002; Knowles et al., 2014). Dha has proven to be a suitable intermediate for side-chain exploration due to its ease of incorporation through a chemical conversion from cysteine mutant precursors, as well as its ability to react bioorthogonally with a vast number of reagents (Bernardes et al., 2008; Chalker et al., 2011; Freedy et al., 2017; Tamura and Hamachi, 2018; Wright et al., 2016a; Yang et al., 2019). Dha has indeed previously been used in a similar manner to enhance enzyme activity (Windle et al., 2017) and has been used in the complementarity-determining region (CDR) loops of a nanobody to create a Boolean logic gates response (Gunnoo et al., 2014).

In this proof-of-concept study, we use a single-domain antibody (Jovčevska and Muyldermans, 2020; Peyvandi et al., 2016), called DesAb-Aβ(3–9), carrying an *in-silico*-designed peptide grafted into the third CDR (CDR3) targeting the N terminus region (residues 3–9) of Aβ42 (Aprile et al., 2017, 2020; Sormanni et al., 2015, 2018). As the aggregation of Aβ42 into amyloid fibrils is one of the fundamental molecular processes underlying AD, the inhibition of Aβ42 aggregation has emerged as a major therapeutic strategy for AD (Knowles et al., 2014). However, since the aggregation of Aβ42 is a complex process consisting of tightly coupled microscopic mechanisms, the perturbation of this process in a haphazard manner can potentially exacerbate the toxicity associated with amyloid formation (Arosio et al., 2014). To address this problem, we use fluorescence-based Aβ42 aggregation assays coupled with a chemical kinetics framework to elucidate the inhibitory mechanism of the different single-domain antibody variants. Thus, we performed two successive rounds of chemical mutagenesis to gain an improvement of five orders of magnitude in inhibiting the primary nucleation rate of Aβ42 aggregation, without compromising the stability of the single-domain antibody.

## Results

**Figure 1:**
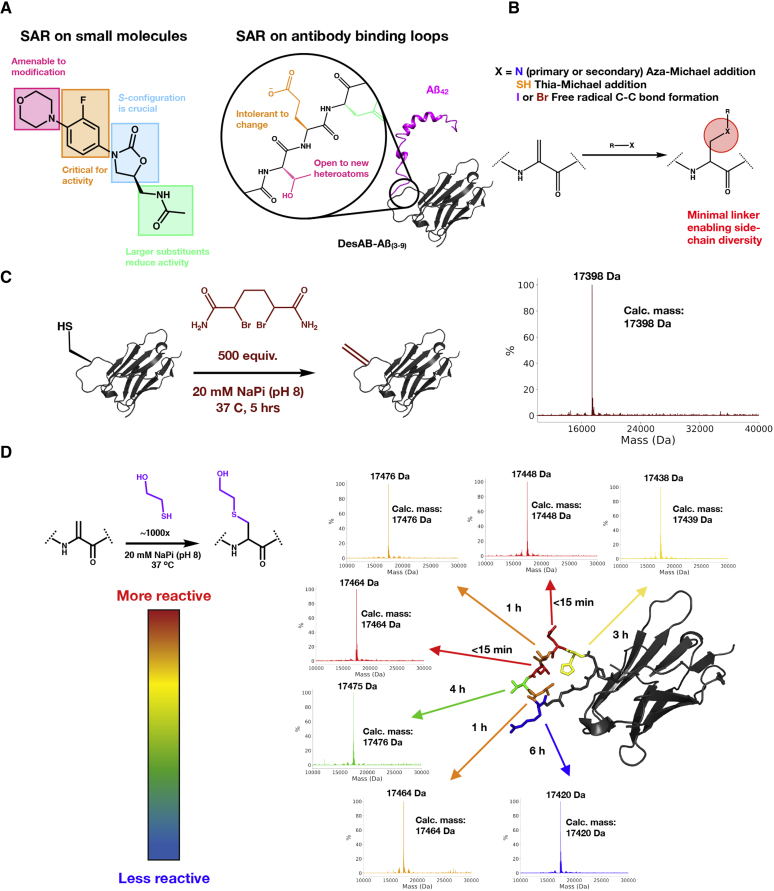
Scanning of the Sequence of the CDR3 Loop by the Post-translational Installation of Dha and Selection of the Three Positions Most Accessible for Chemical Modifications (A) Comparison of traditional SAR on small molecules (here in the case of oxazolidinine as an example) to the SAR on antibody binding loops, as described in this work. (B) Potential avenues for installing side chains at Dha. (C) Dha installation at position T138C, with LC-MS confirming the conversion. (D) Assessment of the accessibility of Dha at the various positions along the CDR3 loop of DesAb-Aβ(3–9) by monitoring the time to completion of the addition of β-mercaptoethanol by LC-MS.

**Figure 2:**
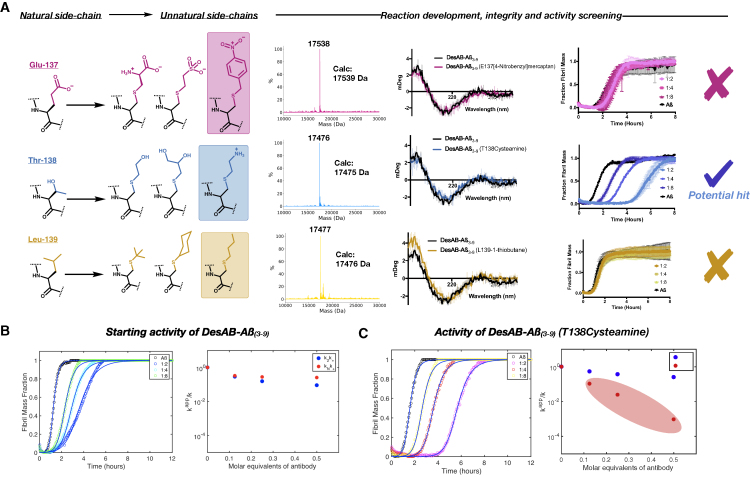
Screen of Panels of Chemical Mutants at the Three Most Accessible Positions in the CDR3 Loop with Non-canonical Side Chains (A) List of the three chemical mutants tested at each of the three positions (E137, T138, and L139) in the CDR3 loop previously selected for their accessibility (Figure 1). We confirmed the chemical modifications by LC-MS, validated their structural integrity after the chemical mutagenesis by circular dichroism (CD), and assessed their potency in inhibiting Aβ aggregation by ThT fluorescence assays at three stoichiometries (1:2, 1:4, and 1:8). The installation of cysteamine at position T138 was selected for further studies. (B and C) Comparison of the aggregation profiles of DesAb-Aβ(3–9) and DesAb-Aβ(3–9) (T138cysteamine); continuous lines represent the fit of the data using the integrated rate law for Aβ aggregation. The scatterplot represents the apparent decoupled primary and secondary nucleation rate constants for each dilution of the antibody.

**Figure 3:**
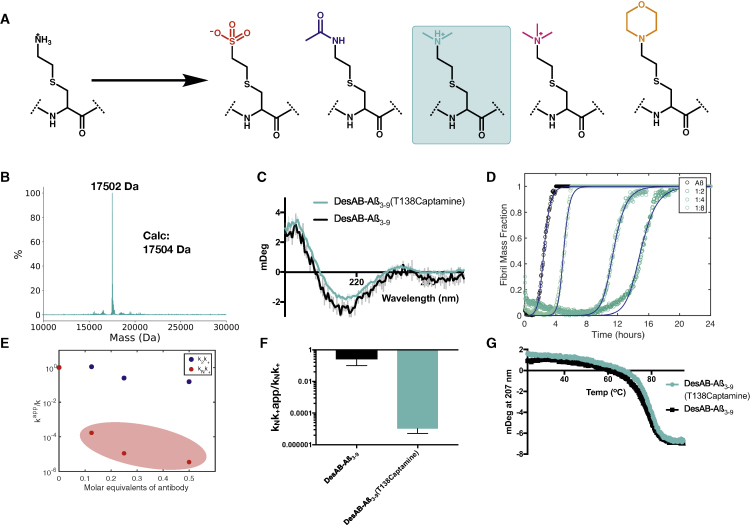
Activity Maturation of DesAb-Aβ(3–9) (T138cysteamine) Using a Panel of Chemical Mutants with Non-canonical Side Chains (A) List of derivatives of cysteamine screened at position T138. (B) LC-MS confirming the addition of captamine to DesAb-Aβ(3–9) (T138Dha). (C) CD of DesAb-Aβ(3–9) (T138captamine). (D) Inhibitory profile of DesAb-Aβ(3–9) (T138captamine); continuous lines represent the fit of the data using the integrated rate law for Aβ aggregation. (E) Scatterplot representing the ratio of the apparent decoupled rates of primary and secondary nucleation with varying equivalents of DesAb-Aβ(3–9) (T138captamine). (F) Comparison of the apparent k_n_k_+_ rate constants of the 1:2 dilutions of DesAb-Aβ(3–9) (black) and DesAb-Aβ(3–9) (T138captamine) (turquoise). (G) The thermal denaturation of DesAb-Aβ(3–9) (black) and DesAb-Aβ(3–9) (T138captamine) (turquoise), as monitored by the CD signal at 207 nm, indicate that the maturation process did not decrease the thermal stability of the starting antibody.

The activity maturation strategy that we describe in this work is based on three steps: (1) we scan the sequence of the CDR3 loop by the post-translational installation of Dha at different positions along the loop and select the positions most accessible for chemical modifications (Figure 1), (2) we screen a panel of chemical mutants with non-canonical side chains at these accessible positions and select the most potent one (Figure 2), and (3) we perform activity maturation on this chemical mutant by further chemical modifications (Figure 3).

### Dha Installation and Accessibility along the CDR3 Loop of DesAb-Aβ(3–9)

Our inspiration for using side-chain exploration as a method for efficient activity maturation for antibodies came from considering how traditional SAR studies are performed on small molecules. More specifically, we aimed to extend to antibodies the precise potentiation by SAR of the desired biological activity of small molecules (Figure 1A). To this end, the non-canonical amino acid Dha is an excellent candidate, since it can be easily installed post-translationally, as an electrophile it is readily and selectively modified by an array of nucleophiles, and importantly for this purpose it has a minimal linking group of just a single carbon (Figure 1B).

Many methods have been explored for the installation of Dha from several amino acid precursors (Bernardes et al., 2008; Chalker et al., 2011). For our efforts here, we chose to rely on the bis-alkylation/elimination of cysteine to Dha using the reagent 2,5-dibromohexadiamide (DBHDA) as this approach has been shown to be effective for Dha conversion on camelid nanobodies previously without the use of added organic solvents (Chalker et al., 2011). With this approach, we started from a previously characterized human VH scaffold whose CDR3 loop was generated by an *in silico* approach to target linear epitopes within intrinsically disordered proteins and peptides, such as Aβ (Figure 1C) (Aprile et al., 2017; Sormanni et al., 2015), and incorporated newly identified thermally stabilizing mutations to promote integrity through the reaction sequences (Julian et al., 2017). To install Dha, single cysteine mutants were created at each of the seven positions along the engineered CDR3 loop, and all seven variants were expressed and purified at yields comparable with that of the starting construct. Conversion of the free cysteine mutants to Dha was first attempted by treating 100-μM solutions of protein (20 mM NaPi at pH 8) with 500 equivalents of powdered DBHDA at 37°C for 5 h (Figure 1C). The reactions were monitored at the end by liquid chromatography-mass spectrometry (LC-MS) for Dha formation. By the end of the 5 h, all positions were observed to undergo complete conversion (>95%) to Dha (Figure 1C), allowing us to explore the potential to subsequently modify each site.

To assess the accessibility of the Dha residue for modification at the seven different positions a benchmark nucleophile, β-mercaptoethanol, was used and the time to completion was closely monitored by LC-MS (50 μM protein, 100 μL total volume, 20 mM NaPi [pH 8], 37°C). This investigation revealed that, while the Dha installation was rather uniform regardless of the position, the rate of the subsequent Michael addition was highly dependent on the local chemical environment (Figure 1D). The time to completion varied as widely as <15 min for certain positions (E137Dha and L139Dha) to 6 h for others (R142Dha) (Figure 1D). One feature that could explain some of the varying rates is the proximity of the Dha residue to negatively charged groups (D and E), which could create unfavorable electrostatic interactions between their carboxylic groups and the attacking thiolate anion. Using these results to guide us, we decided to move forward with three positions along the CDR3 loop based on their accessibility and chemical properties to create an initial panel of three chemical mutants (E137Dha, T138Dha, and L139Dha) for evaluation. Each of these positions were modified by β-mercaptoethanol in an efficient manner and represent three different classes of amino acid side chains, charged, polar, and hydrophobic, respectively. To completely verify that these were indeed the positions modified in this screen, the conjugation sites were confirmed by LC-tandem MS (LC-MS/MS) (Figure S1). While the LC-MS/MS data confirmed the sites of modification, to control that the disulfide bridge within the antibody was not a potential site of Dha formation and subsequent modification, DesAb-Aβ(3–9) was subjected to the same treatment, and no modification was detected.

### Creation and Screening of an Initial Panel of Chemical Mutants

For the creation of an initial panel of chemical mutants, we attempted to explore other conjugation chemistries at Dha beyond the thiol-Michael addition. For example, an aza-Michael addition was initially carried out using piperidine-based reagents (Freedy et al., 2017). While the conjugation with these reagents was efficient, the reagents also led to protein denaturation, likely due to the high pH of the reaction brought about by the excess of the N-nucleophile needed (data not shown). Side-chain installation through radical C-C bond formation was then explored using 2-iodopropane as the initial coupling reagent (Wright et al., 2016a); however, after trying many different conditions with both NaBH_4_ and Zn(0) powder as radical initiators, we never achieved complete conversions (data not shown). We thus decided to use only thiol-based precursors due to their wide availability and their reliable ability to conjugate to Dha.

At each of the chosen positions, a set of three disparate chemical mutants was created and screened for integrity and activity. To start, the chemical mutants at each position largely maintained the overarching physiochemical property of the natural residue that they were substituting (i.e., hydrophobic groups for the L139 position) (Figure 2A). Reaction conditions were kept the same as with the β-mercaptoethanol screen (50 μM protein, 100 μL total volume, 20 mM NaPi [pH 8], 37°C) but the reaction time was optimized for each position. The polar and charged thiol precursors readily modified the various positions to completion, detected by LC-MS, within 1 h. However, the hydrophobic thiols took considerably longer (12 h). Upon completion of the reactions, the chemical mutants were buffer exchanged into standard phosphate-buffered saline and examined for structural integrity by circular dichroism (CD). All of the chemical mutants displayed a CD profile that was fully compatible with the unmodified DesAb-Aβ(3–9) (Figure 2A).

As a next step, we performed activity screening by testing the ability of the variants to inhibit the formation Aβ42 aggregates. The aggregation process was monitored using thioflavin T (ThT), a dye whose fluorescence increases upon binding to amyloid fibrils (Cohen et al., 2013). This protocol yields highly reproducible aggregation curves for Aβ42, and, together with the solution of a system of differential equations that describe amyloid growth (Knowles et al., 2009), it enables the quantification of the microscopic mechanisms underpinning the aggregation that are most perturbed by the presence of an inhibitor. This framework has already led to the identification of numerous small-molecule inhibitors of aggregation, and increased our understanding of the mechanisms of inhibition by endogenous agents, such as molecular chaperones (Arosio et al., 2016; Cohen et al., 2015). To accurately assess the mechanism of inhibition, the ThT-based fluorescence of the aggregation of Aβ42 under reference conditions is carried out alongside a serial dilution of the inhibitor in question. If the inhibitor is indeed active and alters the aggregation profile, these perturbations can be analyzed to elucidate the changes in the different rate constants caused by the inhibitor (Arosio et al., 2014, 2016; Chia et al., 2018; Cohen et al., 2013, 2015; Knowles et al., 2009).

Using this approach, the chemical mutants were screened at three dilutions (1:2, 1:4, and 1:8 [antibody]:[Aβ42]) in triplicate, with the concentration of Aβ42 held constant at 1.5 μM. The aggregation profiles revealed that, while the vast majority of chemical mutations ablated the activity of the antibody compared with the starting wild-type CDR3 loop sequence, the mutant T138cysteamine is a promising potentiating mutation (Figures 2A, S2, and S3). To accurately determine the mechanisms of inhibition, we elucidated the microscopic steps that are most effected by the antibodies by determining the global parameters k_+_k_n_ and k_+_k_2_ (k_+_, k_n_, and k_2_ are the elongation, primary, and secondary rate constants, respectively) by fitting the aggregation curves with the integrated rate laws described above. From the perspective of functional maturation of this single-domain antibody in the context of drug development this evaluation is crucial. For instance, the inhibition of certain microscopic steps can lead to the reduction of amyloid formation at the expense of an increased population of small oligomers, which are increasingly recognized as the most toxic species (Arosio et al., 2014; Chia et al., 2018).

We found that the T138cysteamine variant displays a potentiated ability to inhibit specifically the k_+_k_n_ parameter compared with the unaltered DesAb-Aβ(3–9) (Figures 2B and 2C). We note that, while the resulting cysteamine derivative is very similar to lysine, mutating T138 to lysine using conventional site-directed mutagenesis does not potentiate the antibody activity in the same way as the chemical mutation, even when subjected to the same treatment as the chemically derived cysteamine mutant (Figure S3). This finding could be a result of the fact that addition at Dha creates an epimeric mixture of D and L side chains, or possibly from some subtle conformational changes that the CDR3 undergoes during the transformations. Placing the cysteamine mutation at either of the other positions screened (E137 and L139) also did not have any beneficial effects on the activity of the antibody, confirming that the residue is only potentiating in a specific context (Figure S2).

### Derivatization of T138cysteamine to Amplify Enhancement

Using the cysteamine mutant as an initial guiding point, we sought to further potentiate the activity of DesAb-Aβ(3–9). To do so, we created a panel of derivatives based on the cysteamine scaffold. This panel ranged from subtle physiochemical changes, such as the addition of methyl groups, to more drastic ones, such as the addition of a heterocycle and a charge inversion to investigate the potentiating physiochemical characteristics (Figure 3A). These new chemical mutants were completely amenable to the reaction conditions used to create the initial cysteamine mutant, and all produced correctly folded final products (Figures 3B and 3C). The new chemical mutants were screened in the same manner used previously. The results revealed that one of the derivatives, T138captamine, had a further potentiated activity compared with the starting cysteamine mutant (Figure 3D). Using the kinetic model further shows that the captamine mutant has, again, an enhanced inhibition of the k_+_k_n_ parameter by several orders of magnitude compared with the original DesAb-Aβ(3–9) (Figures 3E and 3F). To accurately assess the individual microscopic steps inhibited by the T138captamine variant, we complemented the aforementioned unseeded kinetic screen with a seeded aggregation reaction to decouple the inhibition of the primary nucleation (k_n_) and fibril elongation (k_+_) reactions. To this end, the 1.5-μM aggregation reactions were also supplemented with 0.45 μM preformed fibril seeds (30% monomer equivalent). With this high concentration of preformed fibril seeds, the contribution of primary and secondary nucleation events to the fibril mass is negligible, and the reaction is purely driven by fibril elongation, thus enabling the investigation of the elongation rate in isolation (Arosio et al., 2014, 2016; Chia et al., 2018; Cohen et al., 2013, 2015; Knowles et al., 2009). Using a 1:1 ratio of Aβ42 monomer to antibody we found that, for both DesAb-Aβ(3–9) and DesAb-Aβ(3–9) (T138captamine), the inhibition of elongation was minimal (Figure S3).

We then characterized the binding of DesAb-Aβ(3–9) (T138captamine) and the starting construct to C-terminally immobilized monomeric Aβ42 using biolayer interferometry (BLI). The results revealed that the two DesAbs bound monomeric Aβ42 under these conditions in a very similar manner (Figure S3). We note that, since it is difficult to apply BLI to accurately quantify the binding to Aβ42, in particular because immobilizing Aβ42 drastically alters the structural ensemble that this disordered peptide adopts compared with its free state in solution, these data do not conclusively explain the mechanism behind the enhanced inhibition, which could also be brought about by the enhanced association with primary oligomers or other aggregated species.

Finally, we measured the conformational stability of our potentiated single-domain antibody DesAb-Aβ(3–9) (T138captamine) by means of thermal denaturation. Our results show that the melting temperature is identical to that of DesAb-Aβ(3–9) within measurement error, and that the melting profiles essentially overlap (Figure 3G). This observation is particularly interesting because, using conventional methods of activity maturation, there is often a significant trade-off between stability and activity when comparing the starting and end constructs (Hoyt et al., 2019), a phenomenon that has been reported to be particularly extreme in the case of aggregation-prone antigens, such as Aβ (Julian et al., 2017). The single uniform melting curve also supports the integrity of the disulfide bond in both the chemical mutant and starting single-domain antibody, as a reduced population yields a multistep melting curve.

## Discussion and Conclusions

Because of advances in chemical biology and synthetic biology, the chemical toolkit for protein engineering has been rapidly increasing over the past decade (Chalker et al., 2011; Hoyt et al., 2019; Krall et al., 2016; Prescher and Bertozzi, 2005; Windle et al., 2017; Wright et al., 2016a). This transformation in protein engineering, and in particular in the technologies aimed at expanding chemical space, has begun to shift methods used to improve protein functionalities toward those used in the realm of the chemistry of small molecules. In the case of antibodies, this strategy has led to some exciting progress, especially in the field of creating peptide-drug conjugates in an ever-improved and rational manner (Rogers et al., 2018).

In this work, we have applied the more subtle technique of chemical mutagenesis to directly optimize antibody activity. We have shown that the CDR3 loop of a single-domain antibody offers accessible sites for modification, with some positions proving more amenable than others based on the local chemical environment. A plethora of diverse side chains could be successfully installed at most sites, and their effects on activity could readily be investigated. This procedure has led us to the identification of an initial potentiated hit, which we then further optimized through derivatization, in a manner like small-molecule SAR studies, culminating with a potentiation of several orders of magnitude over the starting activity. Importantly, such potentiation was finally achieved by the replacement of a single side chain, thus constituting a minimal perturbation of the starting scaffold, which left its thermal stability completely unaffected.

While expanding this strategy to full-length antibodies may require more optimization, Dha installation using cysteine and subsequent modification has already been established to create homogeneous antibody-drug conjugates (Freedy et al., 2017). If Dha installation via cysteine needs to be avoided for some reason, then there are other non-canonical amino acids that can be site-selectively installed to facilitate the use of Dha (Wang et al., 2007).

The chemistry that we have used in this work by no means exhausts the space readily available to chemical mutagenesis. However, a limitation in the system that we have used here is the epimeric mixture of the final non-canonical residue. Although our target was an intrinsically disordered protein, the possibility exists that one of the enantiomers that we have created is more active than the other. Nonetheless, even this relatively small screen was able to yield strongly potentiating non-natural side chains.

Our results, together with those from similar studies not involving antibodies, suggest that many naturally occurring amino acid sequences can be optimized by using replacement with non-canonical side chains (Chalker et al., 2011; Hoyt et al., 2019; Krall et al., 2016; Prescher and Bertozzi, 2005; Windle et al., 2017; Wright et al., 2016a). We thus anticipate that, with the growing toolbox available to expand the chemical space of proteins, maturation by chemical mutagenesis will increasingly be applied in an efficient manner. This approach will provide novel opportunities to substantially improve the activity of antibodies while carrying out relatively modest changes, which will be particularly important in cases where epitope retention is paramount, and also where other essential biophysical properties must be safeguarded.

## Significance

**While powerful methods are available for the discovery of new antibodies, their subsequent development is still a demanding endeavor. To this end, we describe an approach to perform antibody activity maturation based on the use of non-canonical amino acids installed after biosynthesis by chemical mutagenesis. This method greatly expands the chemical space available to antibodies, thus offering a robust route for potency optimization. To illustrate this approach, we show that it can be used to enhance, by five orders of magnitude, the ability of a starting single-domain antibody to inhibit the nucleation step in the aggregation process of the Alzheimer's amyloid-β peptide. These results show that it is possible to perform structure-activity relationship (SAR) studies on antibodies of the type typically performed for small molecules.**

## STAR★Methods

### Key Resources Table

**Table:** 

REAGENT or RESOURCE	SOURCE	IDENTIFIER
**Antibodies**		

DesAB-Aβ(3-9)	This paper	N/A
DesAB-Aβ(3-9)(H136C)	This paper	N/A
DesAB-Aβ(3-9)(E137C)	This paper	N/A
DesAB-Aβ(3-9)(T138C)	This paper	N/A
DesAB-Aβ(3-9)(L139C)	This paper	N/A
DesAB-Aβ(3-9)(T140C)	This paper	N/A
DesAB-Aβ(3-9)(L141C)	This paper	N/A
DesAB-Aβ(3-9)(R142C)	This paper	N/A
DesAB-Aβ(3-9)(T138K)	This paper	N/A

**Bacterial and Virus Strains**		

NEB® 5-alpha Competent *E. coli* (High Efficiency)	NEB	Cat#C2987H
BL21-Gold (DE3) Competent Cells	Agilent	Cat#230132

**Chemicals, Peptides, and Recombinant Proteins**		

Beta - Amyloid (1 - 42) - Lys(Biotin) - NH2, Human	Anaspec	Cat#AS-61484-01
Ampicillin sodium salt	Sigma Aldrich	A9518; CAS: 69-52-3
Isopropyl β-D-1-thiogalactopyranoside (IPTG)	Sigma Aldrich	I6758; CAS: 367-93-1
Imidazole	Sigma Aldrich	I5513; CAS: 288-32-4
Dithiothreitol (DTT)	Sigma Aldrich	D9779; CAS: 3483-12-3
Formic acid	Thermo Fisher	TS-28905; CAS: 64-18-6
Acetonitrile (MeCN)	Sigma Aldrich	900667; CAS: 75-05-8
Iodoacetamide	Sigma Aldrich	I6125; CAS: 144-48-9
Chymotrypsin Sequencing Grade	Roche	Cat#11418467001
2,5-Dibromohexanediamide (DBHDA)	Enamine	CAS: 99584-96-0
2-(bromoethyl)-trimethylammonium bromide	Sigma Aldrich	117196; CAS: 2758-06-7
Potassium thioacetate	Sigma Aldrich	241776; CAS: 10387-40-3
Methanol	Sigma Aldrich	179337; CAS: 67-56-1
Dichlormethane	Sigma Aldrich	320269; CAS: 75-09-2
Hydrochloric acid	Sigma Aldrich	320331; CAS: 7647-01-0
Guanidine hydrochloride	Sigma Aldrich	G3272; CAS: 50-01-1
Ethylenediaminetetraacetic acid (EDTA)	Sigma Aldrich	03609; CAS: 60-00-4
Thioflavin-T (ThT)	Sigma Aldrich	T3516; CAS: 2390-54-7
Bovine serum albumin (BSA)	Sigma Aldrich	Cat#A9418
TWEEN 20	Sigma Aldrich	Cat# P9416
2-Mercaptoethanol	Sigma Aldrich	M6250; CAS: 60-24-2
Zn (0) powder	Sigma Aldrich	324930; CAS: 7440-66-6
Sodium borohydride	Sigma Aldrich	452882; CAS: 16940-66-2
Ammonium acetate	Sigma Aldrich	A1542; CAS: 631-61-8
Piperidine	Sigma Aldrich	104094; CAS: 110-89-4
2-iodopropane	Sigma Aldrich	148938; CAS: 75-30-9
Sodium 2-mercaptoethanesulfonate (MESNA)	Sigma Aldrich	63705; CAS: 19767-45-4
L-cysteine	Sigma Aldrich	30089; CAS: 52-90-4
(4-nitrobenzyl)mercaptan	Sigma Aldrich	755346; CAS: 26798-33-4
Cysteamine	Sigma Aldrich	30070; CAS: 60-23-1
1-thioglycerol	Sigma Aldrich	M2172; CAS: 96-27-5
2-(Dimethylamino)ethanethiol hydrochloride (captamine)	Sigma Aldrich	D141003; CAS: 13242-44-9
N,N,N-trimethylcysteamine	This paper	
N-acetylcysteamine	Sigma Aldrich	363340; CAS: 1190-73-4
2-morpholin-4-ylethanethiol	Santa Cruz Biotechnology	SC-275062; CAS: 4542-46-5
1-Butanethiol	Sigma Aldrich	8.01587; CAS: 109-79-5
2-Methyl-2-propanethiol (Tert-butylmercaptan)	Sigma Aldrich	109207; CAS: 75-66-1
1-Hexanethiol	Sigma Aldrich	234192; CAS: 11-31-9
Amyloid-β (1-42)	This paper	N/A

**Oligonucleotides**		

See table S1	This paper	N/A
Biocytin	Sigma Aldrich	Cat#B4261

**Recombinant DNA**		

DesAB-Aβ(3-9)-pRSET-B vector	This paper	N/A
DesAB-Aβ(3-9)(H136C)-pRSET-B vector	This paper	N/A
DesAB-Aβ(3-9)(E137C)-pRSET-B vector	This paper	N/A
DesAB-Aβ(3-9)(T138C)-pRSET-B vector	This paper	N/A
DesAB-Aβ(3-9)(L139C)-pRSET-B vector	This paper	N/A
DesAB-Aβ(3-9)(T140C)-pRSET-B vector	This paper	N/A
DesAB-Aβ(3-9)(L141C)-pRSET-B vector	This paper	N/A
DesAB-Aβ(3-9)(R142C)-pRSET-B vector	This paper	N/A
DesAB-Aβ(3-9)(T138K)-pRSET-B vector	This paper	N/A

**Software and Algorithms**		

MATLAB R2018a	Mathworks Inc., USA	https://www.mathworks.com/
Kinetic analysis script (MATLAB)	(Arosio et al. 2016)	N/A
GraphPad Prism 8	GraphPad Software	https://www.graphpad.com/scientific-software/prism
Anaconda Python 3.8	Anaconda Inc., USA	https://www.anaconda.com/

**Other**		

EDTA-Free Complete Protease Inhibitor Cocktail Tablet	Roche	Cat#04693159001
HisPur™ Ni-NTA Resin	Thermo Fisher	Cat#88221
ZebaÔSpin Desalting Columns, 7K MWCO, 0.5 mL	Thermo Fisher	Cat# 89882
Oxoid™ Phosphate Buffered Saline Tablets	Thermo Fisher	Cat#BR0014G
Protein low-binding Eppendorf tubes	Sigma Aldrich	Cat# Z666505
Terrific-broth media	Sigma Aldrich	Cat# T0918
Overnight Express autoinduction media	Merk	Cat# 71491-5
96-well Half Area Black/Clear Flat Bottom Polystyrene NBS Microplate	Corning	Cat#3881

### Resource Availability

#### Lead Contact

Further information and requests for resources should be directed to the Lead Contact, Michele Vendruscolo (mv245@cam.ac.uk).

#### Materials Availability

Materials and reagents are available from the authors upon reasonable request.

#### Data and Code Availability

The data generated in this study is available upon request and code for the analysis and computational design is available on http://www-mvsoftware.ch.cam.ac.uk/.

### Experimental Model and Subject Details

All of the single domain antibodies were expressed in *E. coli* BL21 (DE3)-Gold cells grown at 37 ^o^C in liquid media. Aβ42 peptide was expressed and purified from *E. coli* BL21 (DE3) pLysS cells grown at 37°C in liquid media.

### Method Details

#### Generation of Cysteine Mutants and Protein Preparation

Cysteine mutants were generated at various positions along the CDR3 loop of DesAb-Aβ(3-9) by quick-change polymerase chain reaction (PCR) with all primers ordered from Sigma-Aldrich. All antibodies were expressed with an N-terminal hexa-histidine tag from a pRSET-B vector in *E. coli* BL21 (DE3)-Gold strain (Agilent Technologies) and purified as previously described (Aprile et al., 2017). Cultures were grown in modified Terrific Broth media (Sigma-Aldrich) supplemented with ampicillin (100 μg/ml) at 37°C and induced with 1 mM isopropyl β- d-1-thiogalactopyranoside (IPTG) at an optical density (OD) of approximately 0.8 and were then left to express overnight at 30°C. Cells were harvested by centrifugation and re-suspended in standard phosphate buffered saline (PBS) with the addition of one EDTA-Free Complete Protease Inhibitor Cocktail Tablet (Roche) per 500 mL of cell culture and subsequently lysed by sonication. Cellular debris was removed by centrifugation at 15,000 rpm (JA-20 rotor, Beckman Coulter). The cleared lysate was then loaded onto loose Ni^2+^-NTA resin (Thermo Fisher) previously equilibrated with PBS containing 10 mM imidazole. After washing the loaded resin with PBS containing 40 mM imidazole the protein was eluted with buffer containing 200 mM imidazole. The excess imidazole was subsequently removed during size-exclusion chromatography (SEC) using a HiLoad 16/600 Superdex 75 pg column (GE Healthcare LifeSciences, Little Chalfont, U.K.) into PBS, previous to the SEC the protein was incubated with 1 mM dithiothreitol (DTT) for 30 min to reduce any dimers that may have formed. The sequence of the singe-domain antibody used is as follows, with the designed CDR3 loop highlighted in bold and the thermally stabilizing mutation from (Julian et al., 2017) is in red: N terminus- MRGSHHHHHHGMASMTGGQQMGRDLYDDDDKDPKLEVQLVESGGGLVQPGGSLRLSCAASGFNIKDTYIGWVRRAPGKGKEWVASIYPTNGYTRYADSVKGRFTISADTSKNTAYLQMNSLRAEDTAVYYCAAGS**HETLTLR**EEEAAAWGQGTLVTVSSGT -C terminus.

#### Liquid Chromatography–Mass Spectrometry (LC-MS)

Protein LC–MS was performed on a Xevo G2-S TOF mass spectrometer coupled to an Acquity UPLC system using an Acquity UPLC BEH300 C4 column (1.7 μm, 2.1 mm × 50 mm). Water with 0.1% formic acid (solvent A) and 95% MeCN and 5% water with 0.1% formic acid (solvent B) were used as the mobile phase at a flow rate of 0.2 mL/min. The gradient was programmed as follows: 95% A for 0.93 min, then a gradient to 100% B over 4.28 min, then 100% B for 1.04 min, then a gradient to 95% A over 1.04 min. The electrospray source was operated with a capillary voltage of 2.0 kV and a cone voltage of 40 V. Nitrogen was used as the desolvation gas at a total flow of 850 L/h. Total mass spectra were reconstructed from the ion series using the MaxEnt algorithm preinstalled on MassLynx software (v4.1 from Waters) according to the manufacturer’s instructions.

#### Liquid Chromatography with Tandem Mass Spectrometry (LC-MS/MS)

Protein solutions were reduced with DTT, alkylated with iodoacetamide, and subjected to enzymatic digestion with chymotrypsin at 37°C. After digestion, the peptide solutions were pipetted into sample vials and loaded onto an autosampler for automated LC-MS/MS analysis. All LC-MS/MS experiments were performed using a Dionex Ultimate 3000 RSLC nanoUPLC (Thermo Fisher Scientific Inc, Waltham, MA, USA) system and a QExactive Orbitrap mass spectrometer (Thermo Fisher Scientific Inc, Waltham, MA, USA). The separation of peptides was performed by reverse-phase chromatography at a flow rate of 300 nL/min and a Thermo Scientific reverse-phase nano Easy-spray column (Thermo Scientific PepMap C18, 2 μm particle size, 100 Å pore size, 75 μm inside diameter (i.d.) x 50 cm length). Peptides were loaded onto a pre-column (Thermo Scientific PepMap 100 C18, 5 μm particle size, 100 Å pore size, 300 μm i.d. x 5 mm length) from the Ultimate 3000 autosampler with 0.1% formic acid for 3 minutes at a flow rate of 10 μL/min. After this period, the column valve was switched to allow elution of peptides from the pre-column onto the analytical column. Solvent A was water + 0.1% formic acid and solvent B was 80% acetonitrile, 20% water + 0.1% formic acid. The linear gradient employed was 2-40% B in 30 minutes. The LC eluant was sprayed into the mass spectrometer by means of an Easy-Spray source (Thermo Fisher Scientific Inc.). All m/z values of eluting ions were measured in an Orbitrap mass analyzer, set at a resolution of 70,000 and was scanned between m/z 380-1500. Data dependent scans (top 20) were employed to automatically isolate and generate fragment ions by higher energy collisional dissociation (HCD) and 25% normalized collisional energy (NCE) in the HCD collision cell and measurement of the resulting fragment ions was performed in the Orbitrap analyzer, set at a resolution of 17,500. Singly charged ions and ions with unassigned charge states were excluded from being selected for MS/MS and a dynamic exclusion window of 20 seconds was employed. All MS/MS data were converted to mgf files and the files were then submitted to the Mascot search algorithm (Matrix Science, London UK) and searched against a custom database containing the UniProt human database and four sequences relating the 4 proteins which were analyzed. Variable modifications of oxidation (M), deamidation (NQ) and beta mercaptoethanol and a fixed modification of carbamidomethyl were applied. The peptide and fragment mass tolerances were set to 20 ppm and 0.1 Da, respectively. A significance threshold value of p<0.05 and a peptide cut-off score of 20 were also applied.

#### Conversion to Dehydroalanine (Dha)

Cysteine mutants were pre-treated with 100x DTT while shaking at room temperature for 30 minutes, excess DTT was subsequently removed and the protein was buffer exchanged into the reaction buffer (20 mM sodium phosphate buffer (NaPi) at pH 8) by a Zeba Spin desalting column equilibrated with the desired buffer. Protein at concentrations between 75-100 μM was added to 500x solid 2,5-dibromo-1,6-dihexamide (DBHDA, Enamine Ltd.) and left shaking at 37°C for 5 hours. The reaction was monitored by LC-MS and when full conversion to Dha was observed excess DBHDA was removed by centrifugation (5 min, 10,000g, 4°C).

#### General Chemical Mutagenesis from Dha

The generation of chemical mutants from Dha was usually conducted at 50 μM of protein at 100 μL with 1000-500x of the desired chemical mutation precursor in 20 mM NaPi at pH 8. The reactions were monitored by LC-MS until full conversion from Dha to the mutation was observed. Upon completion, the proteins were buffer exchanged back into PBS using a Zeba Spin desalting column. For detailed descriptions of each reaction please contact us for exact conditions.

#### Circular Dichroism (CD)

Circular dichroism (CD) spectroscopy was used to analyze protein secondary structure in solution. Samples were diluted to 5 μM in PBS and CD measurements were recorded using a Chirascan spectrophotometer equipped with a Quantum TC125 temperature control unit (25°C). The data was acquired in a 0.1 cm path length with a response time of 1 s, a per-point acquisition delay of 5 ms and a pre- and post-scan delay of 50 ms. Spectra were averaged over three scans, in a wavelength range from 200 nm to 250 nm, and the spectrum from a blank sample containing only buffer was subtracted from the averaged data. The structural stability of the proteins was analyzed by monitoring the CD signal at 207 nm from 20 to 98°C at a rate of 0.5°C min^−1^. Data points were acquired every 0.1°C with a bandwidth of 1 nm. Analysis of the thermal unfolding curves was performed, assuming a two-state unfolding model.

#### Synthesis of 2-mercaptoethyl-N,N,N-trimethylammonium Chloride

5 g of 2-(bromoethyl)-trimethylammonium bromide (1 eq, 4.88 mmol) was transferred into a 250 mL round bottom flask and dissolved in 25 mL H2O. 3 g of potassium thioacetate (1.3 eq, 26.3 mmol) was added to the previous solution and the mixture was heated to 60°C and stirred overnight. The resulting mixture was concentrated under reduced pressure. The product was extracted by stirring the resulting solid in 100 mL of a solution of [1:1] MeOH/CH2Cl2 at room temperature for 15 min. This process was repeated twice in order to remove KBr. The mixture was filtered and the filtrate was concentrated under reduced pressure. The expected product 2-(acetothioethyl)-trimethylammonium bromide was formed (4.88 g, 97%) and used without purification. 1H NMR (D2O, 400 MHz): d = 2.27 ppm (3H, s, Ac), 3.04 ppm (9H, s, NMe3), 3.13-3.17 ppm (2H, m, CH2), 3.32-3.37 ppm (2H, m, CH2). 13C NMR (D2O, 100 MHz): d = 21.5, 29.8, 52.8, 64.3 ppm. 1.19 g of the previous product was transferred into a 100 mL round bottom flask and dissolved in 10 mL 6M HCl. The solution was heated to 85°C and stirred for 1 h. The resulting solution was concentrated under reduced pressure and dried under vacuum. The product 2-mercatoethyl-N,N,N-trimethylammonium chloride was formed (1.12 g, 94%) and used without purification. 1H NMR (D2O, 400 MHz): d = 2.85-2.90 ppm (2H, m, CH2), 3.07 ppm (9H, s, NMe3), 3.12 ppm (1H, s, SH), 3.43-3.49 ppm (2H, m, CH2). ^13^C NMR (D2O, 100 MHz): d = 16.6, 52.9, 67.8 ppm.

#### Amyloid-β Expression and Purification

BL21 (DE23) pLysS cells containing Abeta 42 PetSac plazimid were grown O/N in 37 degrees using autoinduction media. Abeta42 protein was extracted by 3x sonication of pallet (1'ON/1'OFF)x3 with centrifugation in between (16k RPM for 30 min in 4 degrees). After last centrifugation pallet was resuspended in 8M urea and then diluted to 2M final Urea concentration. Protein solution was mixed with DEAE resin and incubated on ice for 30 min. Resin was applied on Büchner funnel and protein was eluted in steps using vacuum and 10mM Tris 50mM NaCl 1 mM EDTA pH 8.5 & 10mM Tris 100mM NaCl 1mM EDTA pH 8.5 buffers. Eluted fractions were lyophilized for few days and then resuspended in 6M GnCl solution then loaded on SEC (HiLoad 26/600 Superdex 75 pg) column and eluted using 25 mM Na2HPO4 0.2 mM EDTA pH 8.5. Fractions were lyophilized.

#### Amyloid-β Aggregation Assay

Lyophilized Aβ42 peptide was dissolved in 6 M guanidinium chloride (20 mM NaPi, 200 μM EDTA, pH 8) and incubated for 3 h on ice. This solution was then buffer exchanged into buffer containing 20 mM NaPi and 200 μM EDTA at pH 8 by SEC using a Superdex 75 10/300 GL column (GE Healthcare), and the peak belonging to monomeric Aβ42 peptide was isolated and collected in protein low-binding Eppendorf tubes (Corning) on ice. Aggregation reactions were prepared by creating solutions of 1.5 μM Aβ42 in the presence of DesAb derivatives in at ratios from 1:2 DesAb to Aβ42 and decreasing down to 1:8, ThT was added to a final concentration of 20 μM. Samples were loaded by pipette into 96-well half-area plates of black polystyrene with clear bottoms and polyethylene glycol coatings (Corning) (80 μL sample/well). Once all samples were loaded plates were sealed to prevent evaporation. Aggregation reactions were carried out at 37°C under quiescent conditions using a CLARIOstar plate reader (BMG Labtech). ThT fluorescence was measured every 2 min through the bottom of the plate with an excitation filter of 440 nm and an emissions filter of 480 nm. To create Aβ42 fibrils for the seeded assays, 5 μM Aβ42 was incubated at 37°C under quiescent conditions overnight in protein low-binding Eppendorf tubes (Corning). The fibrils were vigorously pipetted up and down before being added to the aggregation reaction to a final concentration of 30% monomer-equivalents. All aggregation reaction conditions were done in triplicate and hits were verified with follow up aggregation reactions again in triplicate.

#### Kinetic Analysis

The time evolution of the total fibril mass concentration, M(t), in the absence of seeds is defined by the integrated rate law

$$\frac{M\left(t\right)}{M\left(\infty \right)}=1-{\left(\frac{B_{+}+C_{+}}{B_{+}+C_{+}e^{kt}}\frac{B_{-}+C_{+}e^{kt}}{B_{-}+C_{+}}\right)}^{\frac{k_{\infty }^{2}}{kk_{\infty }^{∼}}}ae^{-k_{\infty }t}$$

where the parameters are described in detail in previous studies (Arosio et al., 2014; Chia et al., 2018; Cohen et al., 2013). These are functions containing the combinations of the two microscopic rate constants k_+_k_n_ and k_+_k_2_ where k_n_, k_+_, and k_2_ are the primary nucleation, elongation, and secondary nucleation rates respectively. The antibodies can perturb the aggregation reaction by inhibiting one or multiple steps. By applying the above equation to describe the macroscopic profiles we extract the k_+_k_n_ and k_+_k_2_ parameters that best fit the data to enable accurate comparisons of inhibition mechanisms.

#### Biolayer Interferometry (BLI)

A streptavidin biosensor (ForteBio, Menlo Park, USA) was coated with 5 μg/ml monomeric C-terminally biotinylated Aβ42 (AnaSpec, Fremont, USA) in PBS + 0.1% BSA (w/v) + 0.02% TWEEN 20 by overnight incubation at 5°C. Control sensors were coated with the same concentration of biocytin (Sigma). Tips were rinsed before binding experiments by incubation for 1 h at 30°C. The association of the antibodies was monitored at concentration of 20 μM in the buffer previously described for 900 s at 30°C using an Octet Red96 (ForteBio, Menlo Park, USA). The dissociation was monitored by subsequently placing the sensors into a well containing pure buffer for a further 1200 s. The binding of the antibodies to the biocytin coated streptavidin sensors was subtracted from the signals for immobilized Aβ42 to account for non-specific binding. The binding data was analyzed using the Octet software as per the manufacturer’s instructions.

## Supplemental Information

Document S1. Figures S1–S3 and Table S1

Document S2. Article plus Supplemental Information
