# Supplementary material for: Systematic Activity Maturation of a Single-Domain Antibody with Non-canonical Amino Acids through Chemical Mutagenesis
Source: Cell Chem Biol. 2021 Jan 21;28(1):70–77.e5. doi: 10.1016/j.chembiol.2020.11.002 (PMC7837213; doi:10.1016/j.chembiol.2020.11.002)
Supplement: Document S1. Figures S1–S3 and Table S1 [file mmc1.pdf]

Cell Chemical Biology, Volume *28*

Supplemental Information

Systematic Activity Maturation of a Single-Domain Antibody with Non-canonical Amino Acids through Chemical Mutagenesis

Philip R. Lindstedt, Francesco A. Aprile, Pietro Sormanni, Robertinah Rakoto, Christopher M. Dobson, Gonçalo J.L. Bernardes, and Michele Vendruscolo

Supplementary Information for

Systematic Activity Maturation of a Single-Domain Antibody with Non-Canonical Amino Acids through Chemical Mutagenesis

Philip R. Lindstedt[1], Francesco A. Aprile[1], Pietro Sormanni[1], Robertinah Rakoto[1], Christopher M. Dobson[1], Gonçalo J. L. Bernardes[1,2,*] and Michele Vendruscolo[1,*]

[1]Centre for Misfolding Diseases, Department of Chemistry, University of Cambridge, CB2 1EW Cambridge, UK

[2]Instituto de Medicina Molecular João Lobo Antunes, Faculdade de Medicina, Universidade de Lisboa, 1649-028 Lisboa, Protugal

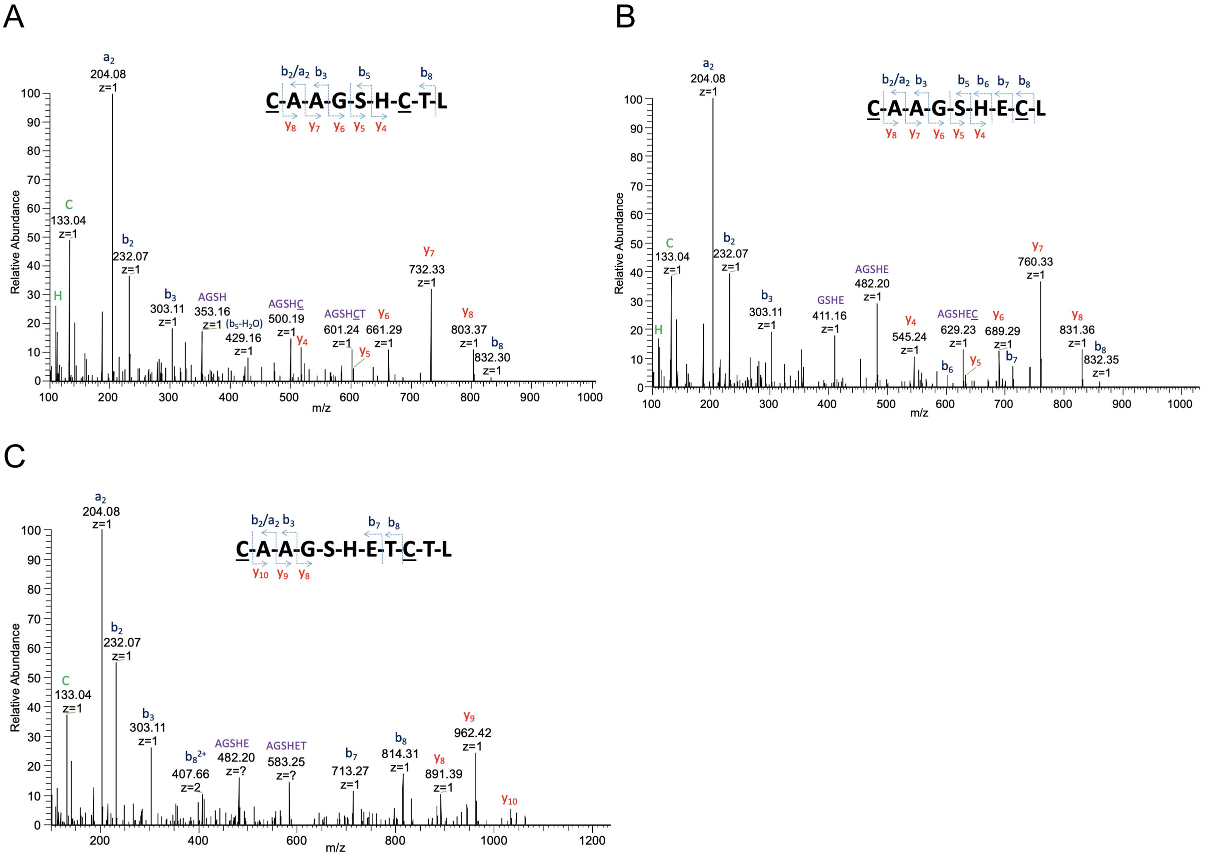

Figure S1. LC-MS/MS spectra confirming β-mercaptoethanol addition sites related to figure 1. (a) MS/MS spectrum of the *m/z* 482.2 doubly charged ion of the chymotryptic peptide CAAGSHCTL from a protein sample containing a carbamidomethyl modification at the N-terminal cysteine residue and a β-mercaptoethanol modification at the cysteine residue at position 137. **(b)** MS/MS spectrum of the *m/z* 496.2 doubly charged ion of the chymotryptic peptide CAAGSHECL from a protein sample containing a carbamidomethyl modification at the N-terminal cysteine residue and a β-mercaptoethanol modification at the cysteine residue at position 138. **(c)** MS/MS spectrum of the *m/z* 597.24 doubly charged ion of the chymotryptic peptide CAAGSHETCTL from a protein sample containing a carbamidomethyl modification at the N-terminal cysteine residue and a β-mercaptoethanol modification at the cysteine residue at position 139.

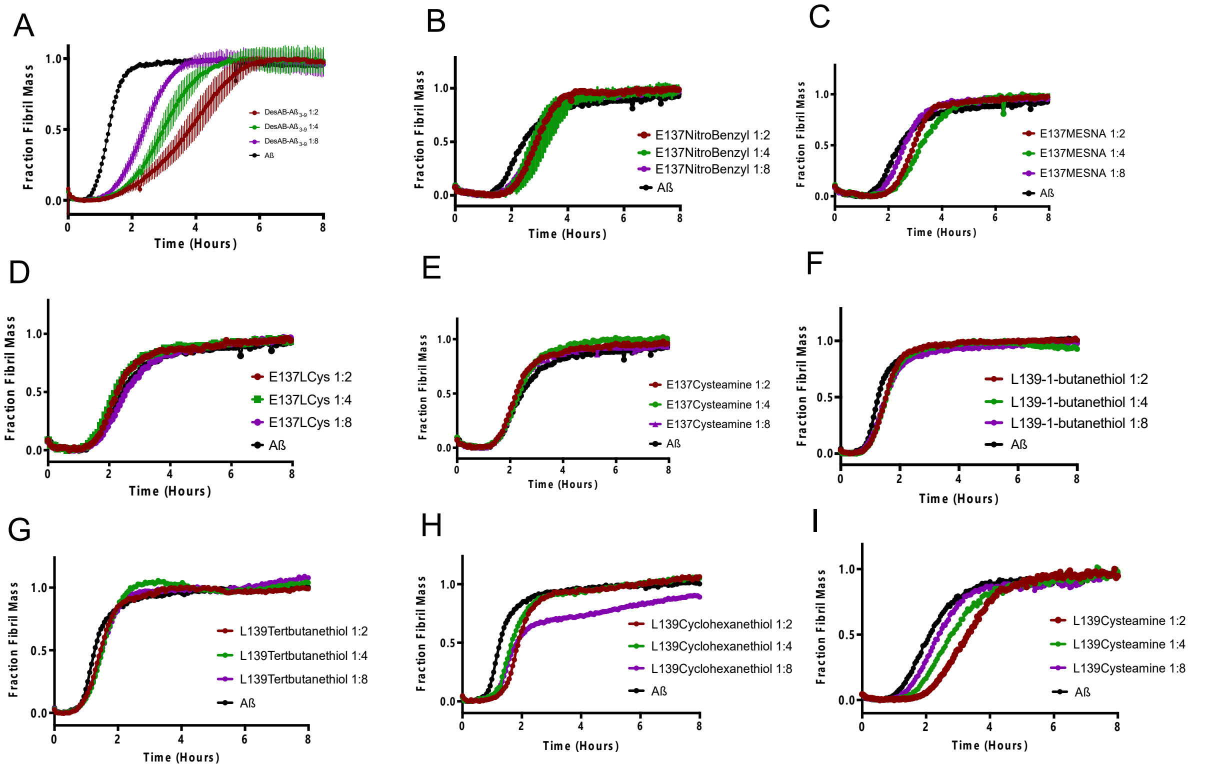

Figure S2. E137 and L139 chemical mutants Aβ42 aggregation assays related to figure 2. (a) Inhibitory profile for the dilution series of the starting engineered antibody. **(b)** Dilution series with the mutant DesAb-Aβ(3-9)(E137[4-nitrobenzyl]mercaptan). **(c)** Dilution series with the mutant DesABb-Aβ(3-9)(E137MESNA). **(d)** Dilution series with the mutant DesAb-Aβ(3-9)(E137LCys). **(e)** Dilution series with the mutant DesAb-Aβ(3-9)(E137cysteamine). **(f)** Dilution series with the mutant DesAb-Aβ(3-9)(T138β-mercaptoethanol). **(g)** Dilution series with the mutant DesAb-Aβ(3-9)(T138-1-thioglycerol). **(h)** Dilution series with the mutant DesAb-Aβ(3-9)(T138cysteamine). **(i)** Dilution series with the mutant DesAb-Aβ(3-9)(T138MESNA). **(f)** Dilution series with the mutant DesAb-Aβ(3-9)(L139-1-butanethiol). **(g)** Dilution series with the mutant DesAb-Aβ(3-9)(L139tertbutanethiol). **(h)** Dilution series with the mutant DesAb-Aβ(3-9)(L139cyclohexanethiol). **(i)** Dilution series with the mutant DesAb-Aβ(3-9)(L139Cysteamine). All aggregation reactions were run in triplicate.

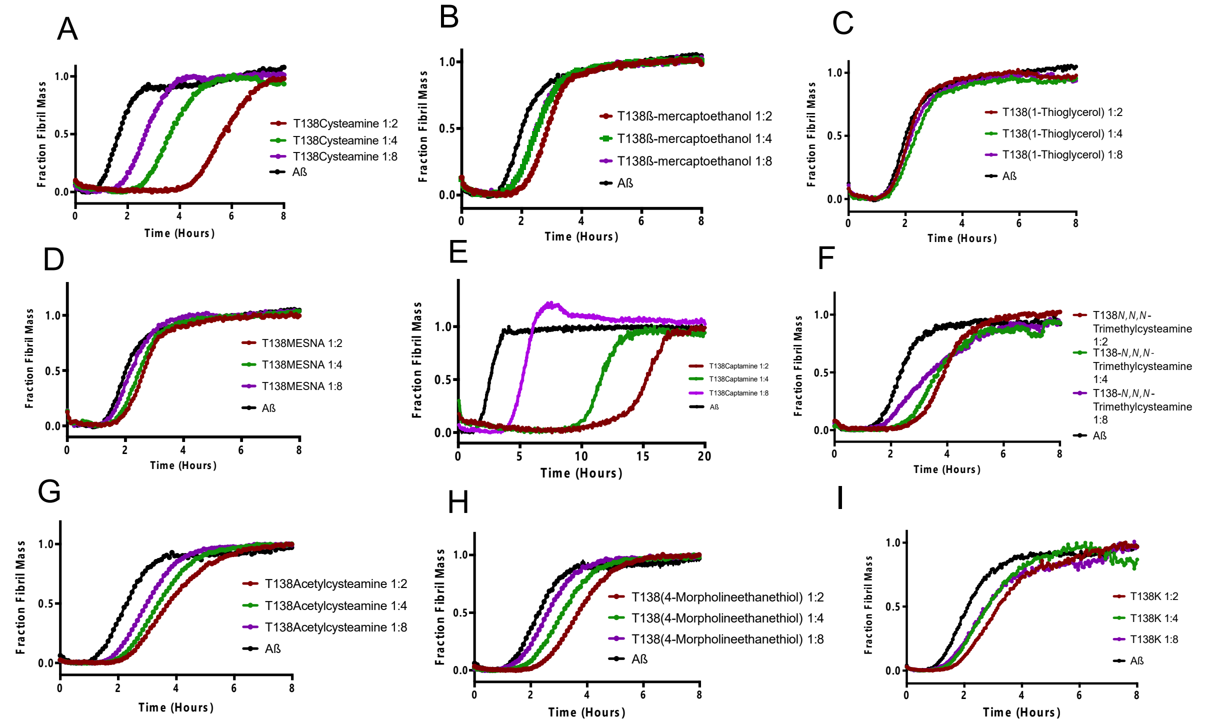

J

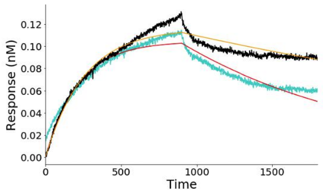

Parameters	DesAB-Aβ$_{3-9}$	DesAB-Aβ$_{3-9}$ (T138captamine)
K_D (M)	1.49E-06 ± 2.85E-08	2.07E-06 ± 3.51E-08
k_{on} ($M^{-1}s^{-1}$)	1.82E+02 ± 2.19E+00	3.84E+02 ± 5.46E+00
k_{dis} (s^{-1})	2.72E-04 ± 4.02E-06	7.95E-04 ± 7.32E-06

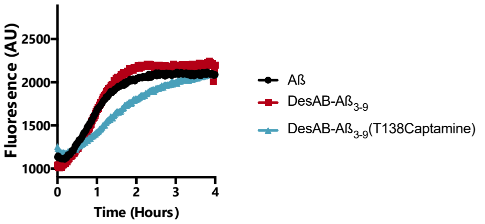

Figure S3. T138 chemical mutants Aβ42 aggregation and binding assays related to figures 2 and 3. (a) Dilution series with the mutant DesAb-Aβ(3-9)(T138cysteamine). **(b)** Dilution series with the mutant DesAb-Aβ(3-9)(T138β-mercaptoethanol). **(c)** Dilution series with the mutant DesAb-Aβ(3-9)(T138-1-thioglycerol). **(d)** Dilution series with the mutant DesAb-Aβ(3-9)(T138MESNA). **(e)** Dilution series with the mutant DesAb-Aβ(3-9)(T138captamine), note the change in the scale of the x-axis. **(f)** Dilution series with the mutant DesAb-Aβ(3-9)(T138*N,N,N*-trimethylcysteamine). **(g)** Dilution series with the mutant DesAb-Aβ(3-9)(T138*N*-acetylcysteamine). **(h)** Dilution series with the mutant DesAb-Aβ(3-9)(T138-4-morpholinethanethiol). **(i)** Dilution series with the mutant DesAb-Aβ(3-9)(T138K). **(j)** BLI data for binding to C-terminally biotinylated Aβ42. **(k)** Aβ42 fibril elongation assay. All aggregation reactions were run in triplicate.

Table S1. List of oligonucleotides related to key resource table

REAGENT or RESOURCE	SOURCE	IDENTIFIER
Oligonucleotides		
Forward primer for H136C mutation: 5'-GGGATCTTGCGAAACCCTGACCCTGCGCGAGGA-3'	This paper	N/A
Reverse primer for H136C mutation: 5'-GGTTTCGCAAGATCCCGCTGCGCAATAATACACAGC-3'	This paper	N/A
Forward primer for E137C: 5'-ATCTCATTGCACCCTGACCCTGCGCGAGGAAGA -3'	This paper	N/A
Reverse primer for E137C mutation: 5'-TCAGGGTGCAATGAGATCCCGCTGCGCAATAATAC-3'	This paper	N/A
Forward primer for T138C mutation: 5'-CATGAATGCCTGACCCTGCGCGAGGAAGAGGCG-3'	This paper	N/A
Reverse primer for T138C mutation: 5'-GGGTCAGGCATTCATGAGATCCCGCTGCGCAATAATA-3'	This paper	N/A
Forward primer for L139C mutation: 5'-TGAAACCTGCACCCTGCGCGAGGAAGAGGCGG-3'	This paper	N/A
Reverse primer for L139C mutation: 5'-CAGGGTGCAGGTTTCATGAGATCCCGCTGCGCAA-3'	This paper	N/A
Forward primer for T140C mutation: 5'-ACCCTGTGCCTGCGCGAGGAAGAGGCGGCC-3'	This paper	N/A
Reverse primer for T140C mutation: 5'-CGCAGGCACAGGGTTTCATGAGATCCCGCTGCG-3'	This paper	N/A
Forward primer for L141C mutation: 5'-CTGACCTGCCGCGAGGAAGAGGCGGCCGC-3'	This paper	N/A
Reverse primer for L141C mutation: 5'-TCGCGGCAGGTCAGGGTTTCATGAGATCCCGCT-3'	This paper	N/A
Forward primer for R142C mutation: 5'-ACCCTGTGCGAGGAAGAGGCGGCCGCGTG-3'	This paper	N/A
Reverse primer for R142C mutation: 5'-TTCCTCGCACAGGGTCAGGGTTTCATGAGATCCC-3'	This paper	N/A
Forward primer for T138K mutation: 5'-GGGTCAGCTTTTCATGAGATCCCGCTGCGCAATAATA-3'	This paper	N/A
Reverse primer for T138K mutation: 5'-CATGAAAAGCTGACCCTGCGCGAGGAAGAGGCG-3'	This paper	N/A
Forward primer for E80K mutation: 5'-GAAAGGCAAGGAATGGGTGGCGAGCATTTATCCGACC-3'	This paper	N/A
Reverse primer for E80K mutation: 5'-CCCATTCCTTGCCTTTCCCGGGTGCACGACGCA-3'	This paper	N/A
